# Medium-term restenosis after carotid endarterectomy by patch type: a single-centre retrospective study comparing biological with synthetic patch materials

**DOI:** 10.1308/rcsann.2024.0097

**Published:** 2025-04-03

**Authors:** M Power Foley, N Doolan, T Connelly, MP McMonagle

**Affiliations:** University Hospital Waterford, Ireland

**Keywords:** Carotid endarterectomy, Patch angioplasty, Vascular graft materials, Restenosis, Neointimal hyperplasia

## Abstract

**Introduction:**

Carotid endarterectomy (CEA) with patch angioplasty is associated with lower restenosis rates compared with primary closure alone. However, evidence regarding patch-material superiority in the mitigation against neointimal hyperplasia and restenosis is limited. This retrospective observational study investigated medium-term restenosis rates between commercially available biological and synthetic carotid patches.

**Methods:**

All primary CEA with patch angioplasty performed between 2007 and 2019 at a single university hospital were identified from theatre records. Restenosis was defined using the European Society for Vascular Surgery duplex criteria, either moderate (50–69%, PSV >213cm/s) or critical (70–99%, PSV >274cm/s). Chi-square tests and Kaplan-Meier curves were used to compare restenosis rates between biological (bovine pericardium) and synthetic patches (Dacron, PFTE and polyester-urethane).

**Results:**

Overall, 127 CEAs were included in the restenosis analysis. Bovine pericardium was the patch material used most frequently (60%, *n*=75). Median follow-up with duplex was 40.0 months (range 0–144). Moderate restenosis was detected in 14 CEAs (11%) and critical restenosis in 10 (7.8%). Compared with synthetic material, bovine was significantly associated with >50% restenosis but not >70% (*p*=0.042 and *p*=0.197, respectively). However, Kaplan-Meier curves demonstrated similar rates of >50% and >70% restenosis between patch types at five years (*p*=0.081 and *p*=0.080, respectively). There was no significant difference in peri-operative complication rates between patch types.

**Conclusions:**

These results indicate medium-term restenosis rates after CEA are similar between biological and synthetic patches. However, well-designed randomised control trials are required to definitively answer the question of which patch material is superior for carotid reconstruction.

## Introduction

Carotid endarterectomy (CEA) is a well-established treatment for stroke risk reduction in patients with significant stenosis (50–99%) of the internal carotid artery (ICA).^1^ However, restenosis of the endarterectomised segment impacts the long-term benefits and cost-effectiveness of CEA, exposing patients to an increased risk of further embolic events and the potential need for surgical reintervention.^2,3^ Restenosis rates post-CEA vary from 5% to 36% in the literature, with rates of recurrent ipsilateral stroke nine times higher in patients with untreated critical (>70%) restenosis.^2–4^ Dyslipidaemia, female sex, diabetes mellitus, chronic kidney disease and continued cigarette smoking have all been identified as independent risk factors for restenosis.^5,6^ Conversely, CEA with patch angioplasty has been shown to significantly decrease rates of restenosis compared with primary closure alone, and is considered international best practice.^7–9^

As such, recent research has focused on identifying which patch materials are most resistant to restenosis. The ideal patch material should be readily available, cost-effective, easily malleable, optimally haemostatic without thrombogenicity, resistant to infection and associated with low rates of restenosis. Vein was favoured initially, as it was free, conformable, antithrombogenic and resistant to infection.^10^ However, the additional requirement for harvesting, and reports of pseudoaneurysm formation and catastrophic patch blow-out has led to the development of synthetic alternatives.^11^

Extended polytetrafluoroethylene (ePTFE) and knitted polyester (Dacron) patches are readily available, with low rates of aneurysmal dilatation; however, prosthetic materials are reportedly more susceptible to infection, less conformable, more prothrombotic and prone to bleeding complications.^12,13^ Studies have identified higher rates of perioperative thrombosis and stroke with Dacron, while ePTFE patches require prolonged haemostasis.^14,15^ Bovine pericardium is a commercially available biological alternative with cost comparable with that of existing synthetic patches. It is easily malleable with good mechanical strength, possesses nonthrombogenic properties and is resistant to suture-line bleeding, as well as being theoretically more resistant to infection.^16–20^ As such, bovine pericardium has steadily gained popularity in vascular surgery as the CEA patch of choice.^9^ However, a recent meta-analysis of randomised and quasirandomised studies comparing autologous vein, synthetic and biological patches concluded that there is a dearth of good quality evidence to conclude which material is superior.^11^

The aim of this single-centre retrospective study was to examine rates of medium-term restenosis after primary CEA between biological and synthetic graft materials.

## Methods

The study design was guided by the STROBE guidelines for observational studies.^21^ Institutional approval from the hospital audit committee was granted for a retrospective chart review. All patients undergoing primary CEA with patch angioplasty performed at a university-affiliated hospital between 2007 and 2019 were identified. The primary indication for CEA was symptomatic 50–99% ICA stenosis identified on preoperative duplex ultrasonography, as determined by the NASCET criteria. Decision to proceed with CEA for asymptomatic 50–99% stenosis was dependent on the individual patient risk profile and varied between consultant surgeons.

All CEAs were performed by a one of five consultant vascular surgeons via conventional longitudinal arteriotomy. The most commonly used patches in our institution were Synovis VascuGaurd (bovine pericardium peripheral vascular patch, Baxter International, IL, USA), Acuseal (ePTFE cardiovascular patch, Gore, DE, USA), Dacron Haemoshield (knitted polyester patch, Boston Scientific, MA, USA) and B-Braun Vascular Patch (polyester-urethane patch, Berlin, Germany). The majority of patients were on best medical therapy (including an antiplatelet agent and statin), which was continued postoperatively. Patients requiring anticoagulation for other indications were commenced on antiplatelets on a selective basis depending on the individual risk profile. All patients were entered into a postoperative surveillance programme with an annual carotid duplex ultrasound as per institutional guidelines.

### Data collection and patient selection criteria

Patients were identified from our institution’s electronic medical records and the Lothian Surgical Audit system. Demographic data included; age, sex, indication for surgery and comorbidities. Operative notes were reviewed individually for surgical technique, patch material used and suture type. Eversion endarterectomies, primary closure post-CEA, carotid reconstruction with an interposition graft, carotid ligation and re-do CEAs were excluded from the study, in addition to patch angioplasty with vein or an unspecified/unidentifiable material.

### Outcomes

Outcomes were reported as per published standards of the Society for Vascular Surgery.^22^ The primary outcome was defined as moderate (50–69%) and critical (70–99%) restenosis after primary CEA with patch grafting and comparison was made between the biological (bovine pericardium) and synthetic (Dacron, ePFTE and polyester-urethane) graft materials. The degree of restenosis was determined using duplex ultrasonography of the ICA. Moderate restenosis >50% was defined as a peak systolic velocity (PSV)>213cm/s and critical restenosis >70% as PSV >274cm/s.^23,24^ Cases where stenotic areas post-CEA were visualised in the common carotid artery proximally or in the native ICA distal to the patch were not considered to be ‘restenosis’. Secondary outcomes included; perioperative stroke, haematoma formation requiring reoperation, patch thrombosis and patch infection.

### Statistical analysis

All data were analysed using Statistical Package for the Social Sciences (SPSS) software Version 27.0 (IBM SPSS Inc., Armonk, NY, USA). Normally distributed continuous data were expressed as mean±standard deviation (SD), while median (range) was used to describe the abnormally distributed continuous data. Categorical variables were presented as count and percent. Time intervals were characterised using median (range) months. Chi-square tests and odds ratio were used to analyse categorical variables. The Mann Whitney *U* test and Kruskal-Wallis test were used to analyse nonparametric data. Kaplan-Meier survival curves were used to assess overall survival and freedom from restenosis at specific time points, censoring patients who died during follow-up and those who were lost to follow-up. The level of statistical significance for null hypothesis testing was *p*<0.05.

## Results

During the study period, 161 carotid reconstructions performed on 142 patients were identified from the database. CEAs utilising eversion technique (*n*=3), primary closure (*n*=10), unplanned ICA ligation (*n*=1), re-do surgery (*n*=5) and three cases where the patch material could not be identified were excluded. Out of 139 primary CEAs with patch angioplasty, 12 had no follow-up imaging and were therefore excluded from the final analysis investigating restenosis rates.

Of the cases lost to follow-up, ten had a documented explanation: three died before scheduled imaging, five were discharged from care, one emigrated and one did not attend scheduled follow-up appointments. The demographics characteristics are outlined in Table 1*.* Almost two-thirds of CEAs (60.8%, *n*=76/125) were performed for symptomatic stenosis. Chi-square analysis was used to compare rates of comorbidities and perioperative details between biological and synthetic patches (Table 2). Notably, proportionally more patients with ischemic heart disease underwent CEA with a bovine patch (*p*=0.044) and shunting was utilised more frequently during CEA with bovine patches (*p*=0.030).

**Table 1:** Demographics, comorbidities, indications for surgery and patch type

	Total		Bovine		Synthetic		*p*
	*n*	%	*n*	%	*n*	%	
Male sex	75/127	59%	43/75	57.3%	32/52	61.5%	0.636
Median age (range)	65.0 years (32.0–87.0)		65.0 years (32.0–87.0)		66.0 years (45.0–84.0)		0.802
Comorbidities							
Hypertension	96/100	96.0%	58/60	96.7%	38/40	95.0%	>0.999
Dyslipidiaemia	104/117	88.9%	65/73	89.0%	39/44	88.6%	0.946
Diabetes mellitus	52/114	45.6%	34/72	47.2%	18/42	42.9%	0.652
Ischaemic heart disease	56/97	57.3%	39/59	66.1%	17/38	44.7%	0.038
Atrial fibrillation	19/97	19.6%	15/59	25.4%	4/38	10.5%	0.071
COPD	32/102	31.4%	24/63	38.0%	8/39	20.5%	0.063
CKD (eGFR <60)	40/120	33.3%	24/74	32.4%	16/46	34.8%	0.791
Active smoker post-CEA	25/95	26.3%	14/60	23.3%	11/35	31.4%	0.387
Pharmacological management post-CEA							
Antiplatelet monotherapy and statin	49/101	48.5%	24/63	38%	25/38	66%	0.041
DAPT and statin	34	33.5%	24	38%	10	26%	
Antiplatelet monotherapy, anticoagulant and statin	14	14%	12	19%	2	5.4%	
Anticoagulant and statin	4	4%	3	5%	1	2.6%	
Indication for surgery							
Symptomatic	76/125	60.8%	47/75	62.7%	29/50	58.0%	0.601
Patch type							
Bovine pericardium	75/127	59.1%					
Polyester urethane	16/127	12.6%
ePTFE	33/127	26.0%					
Dacron	3/127	2.4%
Shunted							
Yes	111/124	89.5%	69/73	94.5%	42/51	82.4%	0.030

CAE = carotid endarterectomy; CKD = chronic kidney disease; COPD = chronic obstructive pulmonary disease; DAPT = dual antiplatelet therapy; eGFR = estimated glomerular filtration rate; ePTFE = extended polytetrafluoroethylene.

Medications recorded at last follow-up were documented. Chi-Square analysis was used to compare frequencies between biological and synthetic cohorts.

**Table 2:** Univariate analysis of association between risk factors and restenosis rates using chi-square tests

Comorbidity	>50% Restenosis	>70% Restenosis
Male sex	0.191	0.202
Hypertension	0.430	0.521
Dyslipidaemia	0.615	0.907
Diabetes mellitus	0.825	0.771
Ischaemic heart disease	0.964	0.302
Atrial fibrillation	0.785	0.687
COPD	0.876	0.697
CKD	0.688	0.815
Smoker	0.632	0.930
DAPT postoperative	0.454	0.858

CKD = chronic kidney disease; COPD = chronic obstructive pulmonary disease; DAPT = dual antiplatelet therapy.

No significant association found between commonly assessed risk factors and any degree of restenosis.

### Rates of carotid patch restenosis

**Figure 1:**
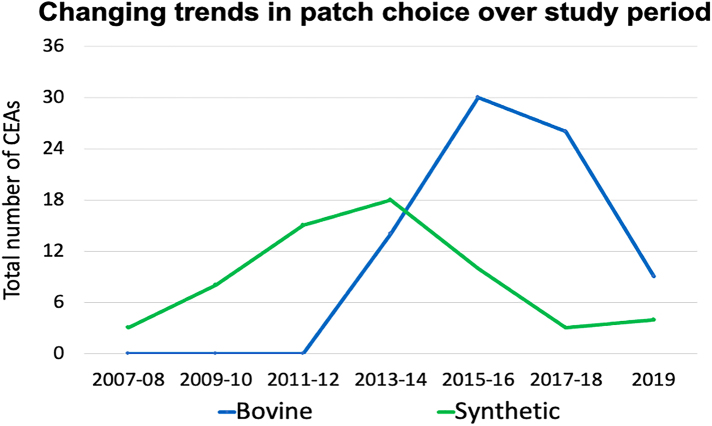
Graph illustrating changing trends in choice of patch material for CEA over the study period. The number of CEAs performed with each patch type during two-year intervals demonstrates bovine surpassing synthetic patches. CAE = carotid endarterectomy.

Overall, 127 CEAs performed on 112 patients were included in the restenosis analysis. Bovine pericardium was the patch material used most frequently (60%, *n*=75). Figure 1 illustrates trends in patch material choice over the study period. The median follow-up with carotid duplex was 40 months (range 1–144) and there was no significant difference in the duration of follow-up between bovine and synthetic patches (40.0 vs 39.0 months, *p*=0.990).

Out of 127 CEAs, 14 (11%) developed >50% restenosis and 10 (8%) developed >70% restenosis during the study period. The median time from surgery to first detection of >50% restenosis on duplex was 17.0 months (range 2–93). Almost two-thirds of restenosis cases (57%, *n*=8/14) were detected within 24 months of surgery. The median time to first detection of >50% restenosis was 21 months for bovine patches compared with 9.5 months for synthetic patches; however, this difference did not reach significance using a Mann-Whitney *U* test (*p*=0.352). Biological patch material was associated with higher rates of >50% restenosis compared with synthetic patches (16% vs 4%, *p*=0.042), but not >70% stenosis (11% vs 4%, *p*=0.197).

**Figure 2:**
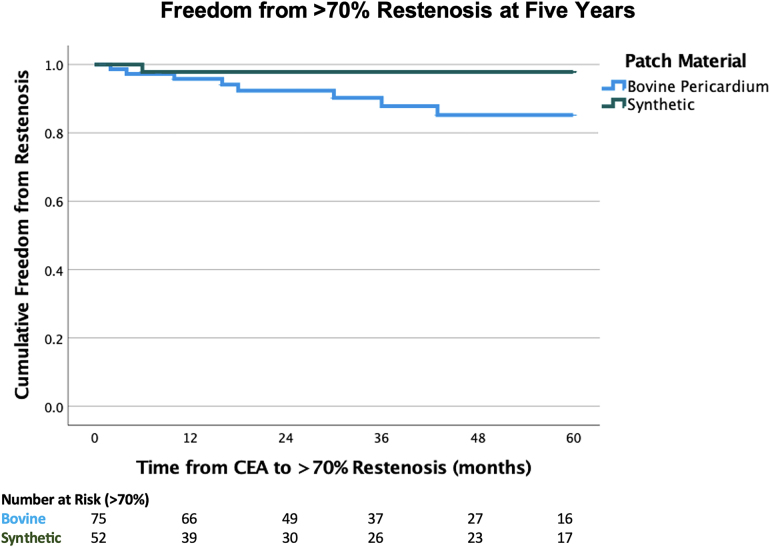
Kaplan-Meier curve illustrating rates of >70% restenosis over time. At five years, 89.3% (*n*=67/75) of bovine pericardial patches were patent compared with 98.1% (*n*=51/52) of synthetic patches (*p*=0.080). CAE = carotid endarterectomy.

**Figure 3:**
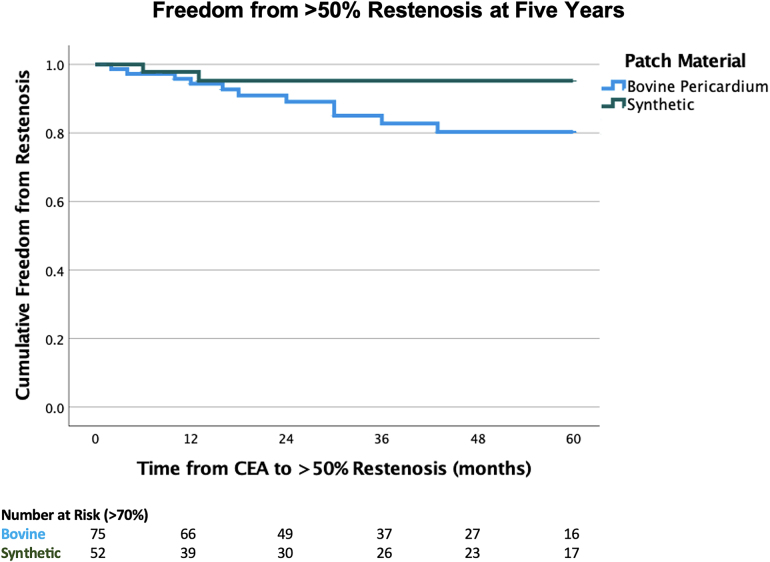
Kaplan-Meier curve illustrating rates of >50% restenosis over time. At five years, 85.3% (*n*=64/75) of bovine pericardial patches were patent compared with 96.2% (*n*=50/52) of synthetic patches (*p*=0.081). CAE = carotid endarterectomy.

Univariate analysis did not demonstrate any difference for rates of restenosis based on patient comorbidities. Kaplan-Meier curves demonstrated no difference in rates of freedom from restenosis between biological and synthetic patches at one, three and five years postoperatively, respectively (Figures 2 and 3).

### Postoperative morbidity and mortality

Information on perioperative morbidity and mortality was available for 139 patients. Three patients (2.1%) developed neck haematomas requiring reoperative intervention and evacuation. One patient required emergency tracheostomy but recovered without long-term sequelae. Two patients (1.4%) developed patch infection; one represented with an infected pseudoaneurysm that required ICA ligation, and the other underwent resection and reconstruction with an interposition vein graft. Two patients (1.4%) developed acute patch thrombosis, one during surgery managed successfully with clot evacuation before skin closure, and one immediately postoperatively. One patient suffered a major ipsilateral ischaemic stroke in the perioperative phase with persistent neurological deficit.

Six CEAs required revision during the follow-up period, of which two were for patch infection and four were for high-grade restenosis considered to be high-risk for stroke. The peak systolic velocities for the four patients who required revision for restenosis was 409cm/s, 386cm/s and 208cm/s, with evidence of flap and soft thrombus; the fourth patient had a magnetic resonance-angiogram instead of duplex ultrasound, which reported >90% stenosis at the ICA origin. All six re-do carotids had bovine patches for their index procedure (*p*=0.036). Notably, high-grade restenosis was associated significantly with the need for revision surgery (*p*=0.007). No significant association were noted between patch types and rates of haematoma, peri-operative cerebrovascular event and patch thrombosis (Table 3).

**Table 3:** Fisher’s exact test (2-sided) comparing complication rates in 2×2 contingency table of bovine vs synthetic patches

Complication	Patch type				*p*
	Bovine		Synthetic		
Any complication	5/78	6.4%	3/59	5.1%	>0.999
Haematoma	2/78	2.5%	1/59	1.7%	>0.999
Stroke	0/78	0%	1/59	1.7%	0.431
Patch infection± pseudoaneurysm	3/78	3.8%	0/59	0%	0.259
Patch thrombosis	0/78	0%	2/59	3.4%	0.184
CEA requiring revision	6/78	7.7%	0/59	9%	*0.037

CAE = carotid endarterectomy.

There were no significant differences in rates of immediate postoperative complications; however, bovine patches were more likely to require revision (*p*=0.037).

**Figure 4:**
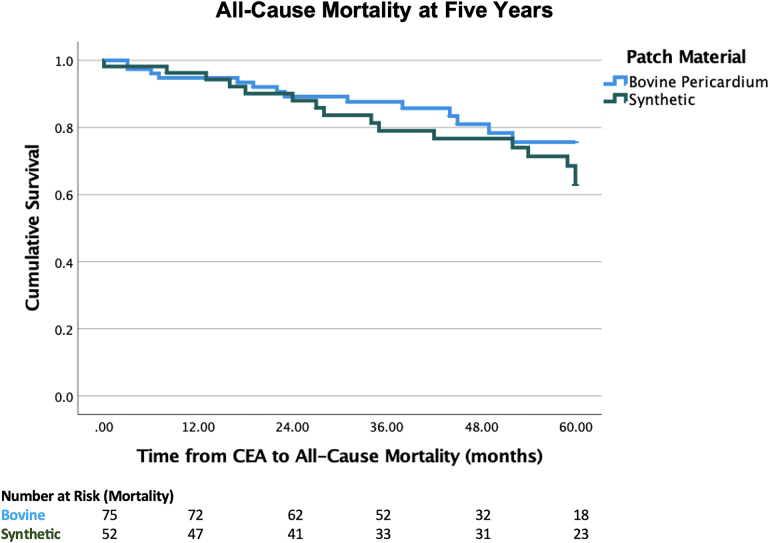
Kaplan-Meier curve illustrating all-cause mortality over time. At five years, 81.8% (*n*=63/77) of bovine pericardial patches were alive compared with 70.9% (*n*=39/55) of synthetic patches (*p*=0.297). CAE = carotid endarterectomy.

The 30-day mortality rate was 0.6% (*n*=1/139). Carotid-specific mortality was 1.4% (*n*=2), including one 30-day mortality and the other was a patient who developed >70% restenosis and subsequently developed a major ipsilateral ischaemic stroke. A further five patients died from contralateral ischaemic strokes during the study period. The all-cause mortality rate to date is 27.3% (*n*=38/139). In addition to the aforementioned stroke-related mortalities, cause of death was available for a further six patients, including four cancer-related mortalities, one ruptured abdominal aortic aneurysm and one cardiac arrest postperipheral bypass surgery. Proportionally fewer patients with bovine patches (20.5%, *n*=16/78) died during the follow-up period compared with synthetic patches (37%, *n*=22/59) (*p*=0.030). Kaplan-Meier curve demonstrated no significant difference in all-cause mortality at five years between patch types (*p*=0.297) (Figure 4).

## Discussion

CEA for stroke risk reduction is a common surgery performed by vascular surgeons. Restenosis at the endarterectomised segment is a well-defined challenge, potentially re-exposing the patient to embolic cerebrovascular events and negating the intended benefit of the procedure. Best published evidence has shown that using patch angioplasty after endarterectomy mitigates against this risk of restenosis compared with primary closure of the vessel.^7,8^ However, determining the optimal patch material for usage remains a focus of ongoing research.

A recent Cochrane Review of 14 trials investigating graft materials for CEA concluded that there was insufficient good quality evidence to answer this question definitively and that further well-designed randomised trials are required.^13^ This single-centre, retrospective study reports on our institutions rates of medium-term restenosis in 127 primary CEAs with patch angioplasty between bovine pericardium (*n*=75) and a pooled cohort of commercially available synthetic patch materials (PTFE (*n*=33), polyester-urethane (*n*=16) and Dacron (*n*=3)). Over the total follow-up period, 11% of all CEAs developed >50% restenosis, comparable with a recent large retrospective study by Liesker *et al*^25^.

### Restenosis rates between biological and synthetic patch materials

Interestingly, we noted a significant association between biological patches and higher rates of >50% restenosis compared with synthetic patches across all follow-up (16% vs 3.8%) and a trend towards higher rates of >70% restenosis (10.66% vs 3.8%). Compared with Liesker *et al*, a similar number of patients with biological and synthetic patches were followed up to five years (Figures 2 and 3).^25^ There was no significant difference in time to restenosis between patch types, as such modulating surveillance protocols by patch material is unlikely to impact detection rates. However, we identified no association between restenosis and previously documented predictive factors, such as female sex, diabetes, chronic kidney disease and cigarette smoking, though the numbers may have been too small to reach significance.^5^

The restenosis rate reported in this cohort is comparable with previously published papers. The largest series to date, including 51,480 CEAs using bovine patches from the Vascular Quality Initiative database, reported 1.3% of cases developed >80% restenosis at one year. However, the outcomes were limited to short-term follow-up and the authors did not define the threshold velocities for restenosis.^9^ Similarly, Oldenburg *et al* reported a ten-year >50% restenosis rate of 11% in 680 bovine patches without defining threshold velocities.^20^

The variability in duration of follow-up and restenosis criteria across studies is a notable weakness among the available literature on the use of bovine pericardium in carotid surgery and makes directly comparing results challenging. In particular, the lack of a consensus criteria for defining restenosis could potentially lead to comparative under- or overestimation of restenosis between studies. This study’s criteria for defining ‘moderate’ restenosis (PSV >213cm/s) was higher than in most previous publications, where thresholds for 50–69% restenosis ranged from PSV 125–140cm/s.^19,26–29^ However, the secondary analysis of the CREST trial reported 10.5% of 1,184 CEAs developed >50% restenosis rate using similar velocities (>210cm/s), although the authors did not differentiate between patch materials.^6^

The ‘critical’ restenosis definition of PSV >274cm/s utilised in this paper is on par with the 250–325cm/s range reported in published studies.^19,26–31^ Hopefully, as the recently updated 2023 ESVS clinical practice guidelines provided diagnostic criteria for >50% and 70% restenosis on duplex ultrasound, further studies will produce more homogenous and comparable results.^23^

The burden on patients and healthcare systems created by high-grade carotid restenosis must also be considered. The majority of 50–69% restenosis can be managed medically, as was the case in this cohort, as long as they remain asymptomatic. However, >70% restenosis was clinically significant and carried higher risks of further thromboembolic events and the need for surgical re-intervention.^2–4^ Out of the ten carotids that developed critical restenosis in this study, one had an major ipsilateral stroke and died. There were no late major ipsilateral strokes in patients with 50–69% restenosis. Furthermore, of the six CEAs that required surgical revision during the study period, four were for high-grade restenosis (*p*=0.007). Interestingly, all of the re-do surgeries were for patients who had bovine patches at initial CEA (*p*=0.037).

### Complication rates between patch materials

Rates of significant perioperative complications were minimal in our study and largely consistent with the existing literature.^9,31^ Interestingly, none of the three patients with bleeding complications occurred with PTFE patches and, although bovine pericardium is hypothesised to be more resistant to infection than prosthetic patches, it was the only material to result in serious infective complications in this cohort. This may be an artefact of bovine pericardium being the most frequently used patch type and unrelated to its material properties.

### Study limitations

The main limitations of this study are its small size and retrospective nature. As data were not recorded prospectively with the exact needs of this study in mind, all the necessary variables were not always available in the medical records. The overall small sample size and variability of patch materials used impacts the statistical power and our ability to draw conclusive associations from the data. Furthermore, the schedule for follow-up duplex imaging was fragmented across the study period due to the lack of standardised surveillance protocol and scarce radiology resources. As the focus was biological versus synthetic, comparative outcomes between synthetic patches were deemed to be outside the scope of this study.

## Conclusion

In this single-centre retrospective study of medium-term patch restenosis after CEA, proportionally more bovine patches demonstrated both >50% and >70% restenosis at five years compared with synthetic patches. Of the ten patients who developed critical restenosis, one went on to have a major ipsilateral stroke and three required high-risk re-do carotid surgery, confirming there is a clinical burden associated with this phenomenon. The results suggest bovine pericardium may be more susceptible to restenosis and infection than previously reported. While this study adds to the growing literature on different patch materials for CEA, we feel it further emphasises the need for well-designed randomised trials to investigate medium- to long-term outcomes and to determine which patch material to use.
